# Effectiveness of Botulinum Toxin on Hand Tremor Intensity and Upper Limb Function in Patients with Parkinson’s Disease: Results of a Systematic Review

**DOI:** 10.5334/tohm.773

**Published:** 2023-08-24

**Authors:** Fariba Eslamian, Neda Dolatkhah, Leila Fallah, Fatemeh Jahanjoo, Vahideh Toopchizadeh, Mahnaz Talebi

**Affiliations:** 1 Physical Medicine and Rehabilitation Research Center, Tabriz University of Medical Sciences, Tabriz, Iran; 2 Faculty of Medicine, Tabriz University of Medical Sciences, Tabriz, Iran; 3 Physical Medicine and Rehabilitation Research Center, Aging Research Institute, Tabriz University of Medical Sciences, Tabriz, Iran; 4 Heidelberg Institute of Global Health (HIGH), University of Heidelberg, Heidelberg, Germany; 5 Neurosciences Research Center, Tabriz University of Medical Sciences, Tabriz, Iran

**Keywords:** Botulinum toxin, Parkinson’s disease, the unified Parkinson’s disease rating scale, Tremor

## Abstract

**Background:**

Hand tremor is a common symptom of Parkinson’s disease (PD). Tremors may be resistant to drug treatments. Therefore, Botulinum toxin (BoNT) could be a good alternative. This study aimed to review and analyze studies on the efficacy and safety of BoNT injection in hand tremor intensity and upper limb function in patients with idiopathic PD.

**Methods:**

A comprehensive search was conducted for studies on the effect of local BoNT injections on tremors in PD patients from 1990 to December 2021. Electronic databases such as Cochrane Central Control Records, PubMed, Scopus, Web of Science, EMBASE, Google Scholar, Clinicaltrial.gov, ProQuest, Science Direct, CINAHL, and Psychoinfo were searched systematically.

**Results:**

Ten studies, comprising one double-blinded randomized clinical trial and nine pilot open-labeled studies with 131 participants, met eligibility criteria. The reported tremor intensity ranged from 1 to 3, and the average tremor duration of 5.93 ± 2.08 years. The injectable dose was 68–100 units of onabotulinum-toxin-A in each upper limb muscle, mostly wrist flexors. The results showed a decrease in unified Parkinson’s disease rating scale (UPDRS)_20 and UPDRS_21 indices by 1.22 ± 1.1 and 1.20 ± 0.9, respectively, without causing severe side effects. The BoNT relative effectiveness in the forearm and arm muscles was reported 6–16 weeks after injection.

**Discussion:**

The kinematic, electromyography-guided, and electrical stimulation evaluations allow for accurate muscle localization and minimize the possibility of BoNT diffusion and antibody formation. More extensive randomized clinical trials with uniform measurement criteria are recommended to reduce bias and provide more accurate conclusions.

**Highlight:**

Tremor treatment in Parkinson’s-disease (PD) is challenging. Drugs effect is temporary, and surgery is critical management. This study reviews the Botulinum-toxin injection efficacy in hand tremor intensity and upper limb function. The results showed a decrease in unified Parkinson’s disease rating scale (UPDRS)_20 and UPDRS_21 by 1.22 ± 1.1 and 1.20 ± 0.9, respectively, 6–16 weeks after injection.

## Introduction

Parkinson’s disease (PD) is the second most common neurodegenerative disorder after Alzheimer’s disease worldwide, which eventually leads to certain movement disorders such as postural instability, bradykinesia, rest tremor, and rigidity [1]. Among these features, hand tremor is the most common disorder [2,3].

PD patients usually have a 4–6 hertz frequency rest tremor, but the “re-emergent tremor” is more troublesome because it interferes with the patient’s ability to hold objects [4]. The tremor may be drug-resistant or require high drug doses, which can cause motor fluctuations [5,6]. So, treatment of the tremor in PD can be challenging.

The effect of usual drugs is temporary, and the patient has to use the drug daily, even up to several times a day. As a result, the patient is exposed to the side effects of medications daily [7]. Cognitive impairment and urinary retention due to the drugs are difficult to tolerate, especially in older patients. For a few people with severe disabilities, surgical treatments may be considered [8].

Botulinum toxin (BoNT), produced by clostridium Botulinum, is one of the most widely applied therapeutic drugs in clinical medicine in patients with motor disorders, pain disorders, and autonomic dysfunction [9,10]. Bacterial strains produce seven distinct neurotoxin antigens called A-G [11]. BoNT type A (BoNT-A) is the common serotype administered and considered an effective treatment for movement and other non-neurologic disorders [12]. Commercial brands of toxins are well-recognized to be valid and safe [13]. Different excipients and diluents are applied in the preparation of the commercial BoNT administered in Europe, Onabotulinumtoxin A (Ona-BoNT/A), Incobotulinumtoxin A (Inco-BoNT/A), and Abobotulinumtoxin A (Abo-BoNT/A)). A general estimation considers 1 unit of Ona-BoNT/A at 1 unit of Inco-BoNT/A and 2.5–3 units of Abo-BoNT/A. However, different clinical situations can alter the equivalence [14].

Various studies have been conducted worldwide concerning the effect of BoNT on tremors among patients with movement disorders and PD [15,16,17]. However, there have been contradictions in the findings, and no systematic review exists regarding the BoNT injection in the upper limbs tremors. The present study was designed to systematically review and update the existing knowledge in identifying clinical aspects, anatomical issues, and associated factors regarding the effect of BoNT on hand tremor management. We sought to suggest an approach to determine the BoNT injection optimum dose, its safety/efficacy, and the appropriate technique in patients resistant to conventional drug therapy.

## Methods

This study reviews cross-over double-blinded randomized clinical trials and pilot studies systematically.

### Literature Search

For accessing relevant studies, the search process was performed using inclusion and exclusion criteria and the medical subject headings (MESH) and keywords, including “Parkinson’s Disease”, “Parkinsonism”, “Idiopathic Parkinson”, “Functional Parkinsonism”, “Treatment”, “Medical treatment”, “Local Botulinum toxin injection”, “Botulinum toxin A injection”, “Botulinum neurotoxin”, “Botulinum toxin-A therapy”, “Incobotulinum toxin”, “Sensor guide Botulinium toxin injection”, “Electromyography guided Botulinium toxin injection”, “Kinematic guided Botulinium toxin injection”, “Kinematic guided botulinum toxin-A treatment”, “Improvement”, “Management”, “Effectiveness”, “Efficiency”, “Ability”, “Debilitating upper limb”, “Hand tremor intensity”, “Upper limb function”, “Kinematic”, “Tremor composition”, “Upper limb accelerometer”, “The Reliability of the Scale”, “Quality of life for Essential Tremor Questionnaire”, “Quality of life”, “Baseline hydraulic hand dynamometer”, “Activities of Daily Living Scoring”, “Fahn-Tolosa Marin Scale”, “Marin tremor rating scale”, “Unified Parkinson Disease Rating Scale”, “Tremor analysis”, “Tremor measures”, “Non-voluntary/involuntary activity of hand”, “Essential tremor”, “Parkinsonian tremor”, “Tremolus movement”, “Electro goniometer”, and the “AND” and “OR” operators using the Cochrane Central Register of Controlled Trials, MEDLINE (PubMed), Scopus, Web of Sciences, EMBASE, Google Scholar, Clinicaltrial.gov, Science Direct, CINHAL, PsychoInfo, and ProQuest databases published during the years 1990 and December 2021.

At the same time, two authors manually searched the literature to identify any possible studies. Endnote software (Thomas Reuters Philadelphia, PA) version 20 was used to manage the findings identified by the mentioned strategies. The two researchers independently conducted a preliminary review of the titles and abstracts, followed by a complete evaluation of the texts of studies to identify eligible studies. The collected articles were reviewed separately to avoid any duplication. In case of disagreement between the two researchers at each stage of selection and evaluation, the third researcher was assigned, and a group discussion was held.

The present study was designed according to the Cochrane Handbook for Systematic Reviews of Interventions [18] and the preferred reporting items for systematic reviews and meta-analyses statement [19]. The PICOS formula (participants, interventions, comparison, outcomes, study design) was applied to determine study inclusion criteria (Table 1). The present study was approved by the Research Council of the Research Center for Evidence-Based Medicine and the Regional Ethics Committee (code IR.TBZMED.VCR.REC.1398.373).

**Table 1:** Eligibility criteria for including studies in systematic review based on PICO.


DOMAIN	DETERMINED CRITERIA

Population/participants	Patients with a family history or clinically diagnosed idiopathic Parkinson’s disease (stage 1 to 3 according to H&Y scale) and hand tremor (uni/bilateral),which one year passed from the definitive diagnosis of their illness and are under pharmacotherapy. There was no restriction on age and sex.

Interventions	Guided Botulinum toxin injection with no restriction on dosage (e.g., high, low) and injection intervals (e.g., daily, weekly)

Control/comparison	Placebo/No treatment

Outcomes	Any beneficial effect of Botulinum toxin on the symptoms of the disease

Study design	Case report, case series, cohort studies, and trials published in English between 1990 and September 2021 with full text available regardless of location


**Abbreviation** H&Y: Hoehn and Yahr.

### Inclusion criteria

All human English-written studies investigating the efficacy of guided BoNT injections on the hand tremors in stages I-III Hoehn &Yahr (H&Y) PD patients for one year after their definitive diagnosis were included. We applied no restrictions regarding the publication status of the studies (Table 1).

### Exclusion criteria

The exclusion criteria included lack of access to the article’s full text, articles published before 1990, studies with not enough quality, reprinting using the information of the same sample, in vitro and animal, and review studies, case report studies with less than five people, study protocols, and letters to the editor. Studies in which the study population included patients with cognitive impairment or psychosis, secondary parkinsonism, a history of BoNT or any other injection during the past six months, a history of surgery, and stroke were also excluded. The results of each study analysis were included for clinical purposes when possible. Otherwise, the available data (for example, when only the analysis results were reported according to the protocol) were used. Regarding the quantitative data, mean and standard deviation were obtained. If these indices were unavailable, they would be reported using the indicators of the central tendency and dispersion (e.g., median and range of changes).

### Data collection

Three authors extracted data from all eligible studies and summarized them. Data contained the author’s name, year of publication, country, design, population, intervention, control, outcomes, sample size, age of participants, evaluation time, results, side effects, and level of evidence (Table 2).

**Table 2:** Characteristics of double-blind and open-label studies to evaluate botulinum toxin injection in the treatment of Parkinson’s disease tremor.


REF NO.	STUDY	YEAR	COUNTRY	STUDY TYPE	POPULATION (DIAGNOSIS)	NUMBER OF PATIENTS WITH PARKINSON	NUMBER OF DROPPED OUTPATIENTS	NUMBER OF INCLUDED PATIENTS	AGE (MEAN ± SD)	SEX	DURATION (YEAR)	PD SEVERITY (H & Y)	MAXIMUM FOLLOW UP (WEEK)	ASSESSMENTS TIME POINTS (WEEK)	OCEBM LEVEL OF EVIDENCE

20	Samotus et al.	2017	England	Open label	24 ET, 28 PD	28	14	14	65.5 ± 11.5	21 F7 M	7.5 ± 3.1	UC	96	0, 6, 16, 22, 32, 38, 48, 54, 64, 70, 80, 86, 96	III

21	Pullman	1996	USA	Open label	91 FD, 28 SD, 17 GD, 107 UED, 29 LED, 17 ET, 15 PD, 5 CBLT, 2 CTS, 6 CIS, 5 DDS, 1 PLSS	15	UC	15	63.9	UC	UC	1 to 2.5	384	6 to 8	III

22	Rahimi et al.	2013	England	Open label	7 PD	7	0	7	59 ± 7.7	5 F2 M	5.6 ± 3.1	UC	12	4, 8, 12	III

23	Rahimi et al.	2015	England	Open label	28 PD	28	Week 6: 3Week 32: 3After week 32: 4	18	65.5 ± 11.5	21 F7 M	7.5 ± 3.1	2 to 3	32	0, 6, 16, 22, 32	III

24	Mittal et al.	2017	USA	Cross over double blind RCT with placebo control	30 PD	34:IncoA-Placebo: 16Placebo-IncoA: 18	4:IncoA-Placebo: 2 (moved)Placebo-IncoA: 2 (health problem)	30:IncoA-Placebo: 14Placebo-IncoA: 16	67.12	23 F7 M	3.12	UC	8	0, 4, 8	I

25	Jankovic & Schwartz	1991	USA	Open label	14 DysT, 12 ET, 22 Dyst+ET, 1PD, 1 PI, 1 MB	1	0	1	UC	UC	UC	1 to 3	UC	1	IV

26	Trosch & Pullman	1994	USA	Open label	14 ET, 12 PD	12	0	12	66.3	UC	UC	1 to 3	6	3, 6	III

27	Henderson	1996	Sweden	Open label with placebo control	17 non-DysT: 3 PD, 2 PD+ET, 3 MSA, 1 MD, 4ET, 2 ET + mild EPS, 1 HT, 1 EPT	3	0	3	76.3 ± 9.46	2 F1 M	5	Moderate to severe	6	0, 4, 8	III

28	Niemann & Jankovic	2018	USA	Retrospective cohort	99 ET, 6 PD, 37 DysT, 1 COT	6	0	6	UC	UC	UC	UC	120	0, 120	III

29	Kreiser	2019	France	Retrospective cohort	21 ET, 8HTFbl, 4 Dyst, 4 Pwt, 1 PD	1	0	1	UC	UC	UC	UC	4	0, 4	IV


**Abbreviation** PD: Parkinson’s disease; H&Y: Hoehn and Yahr; OCEBM: Oxford center for evidence-based medicine; DysT: Dystonic tremor; ET: Essential tremor; PI: peripherally included; MB: midbrain; FD: focal dystonia, SD: segmental dystonia, GD: generalized dystonia, UED: upper extremity dystonia, LED: lower extremity dystonia, CBLT: Cerebellar tremor; CTS: cerebral trauma spasticity, CIS: cerebral infarct spasticity, DDS: dermyelinating disease spasticity, PLSS: primary lateral sclerosis spasticity, MSA: Multiple system atrophy; EPS: Extrapyramidal symptoms; HT: Head titubation; EPT: exaggerated physiological tremor; COT: Cerebellar outflow tremor; HTFbl: Holmes tremor secondary to a focal brain lesion; Pwt: Primary writing tremor; IncoA: Incobotulinumtoxin A; F; female; M; Male; IncoA: Incobotulinumtoxin A; UPDRS: Unified Parkinson’s disease rating scale; NIHCGC tremor severity: National Institutes of Health Collaborative Genetic Criteria; PGIC: Patient Global Impression of Change Scale; PDQL: Parkinson disease quality-of-life questionnaire; FTM: Fahan-Tolosa-Martin tremor scale; UC: unclear.

## Results

### Study selection process

**Figure 1:**
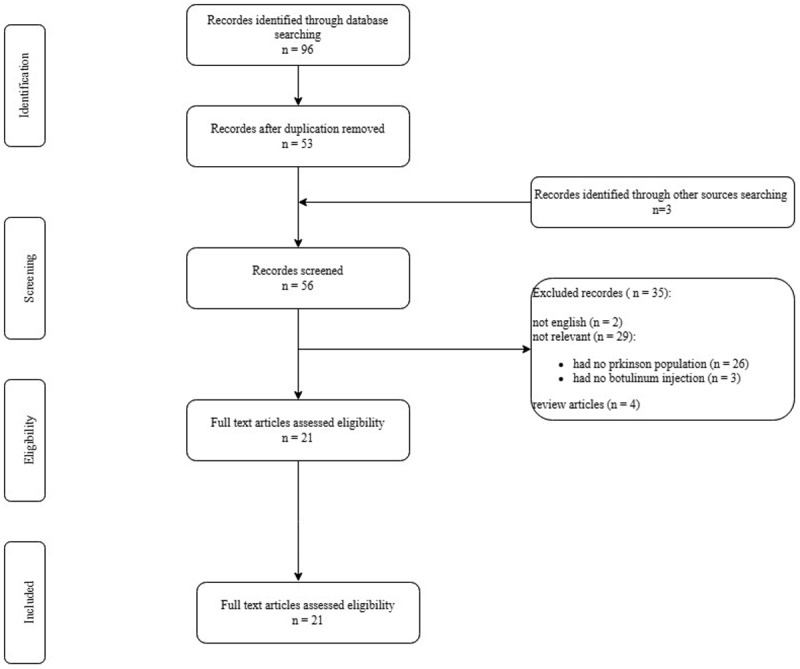
PRISMA flow chart summary of the systematic search process.

A thorough review of the collected data showed that ten articles were eligible for further review (Figure 1) [20,21,22,23,24,25,26,27,28,29].

### Study Characteristics

After a more detailed review, no study was excluded at this stage (Table 2). These ten studies included one cross-sectional double-blinded, randomized clinical trial [24]. The other nine studies included single-center open-labeled randomized pilot studies classified in the cohort study category. These studies were conducted between 1990 and 2021, five in the United States [25,26,27,28,29] and three in the United Kingdom [20,22,23]. In most studies [20,21,25,26,27,28,29], the etiology of the patient tremor had not been addressed, and only three articles specifically examined PD tremors [22,23,24].

One hundred and thirty-one patients were studied. The mean ± standard deviation of patients’ age was 66.23 ± 5.18 years. Sex and duration of hand tremors were reported in 5 and 4 studies, respectively. Of the 96 patients whose sex was reported, 72 (75%) were male. The mean ± standard deviation of hand tremor duration was 5.93 ± 2.08 years. Patients’ tremor intensity ranged from 1 to 3 on the 1–4 scoring system.

### Results of the muscle selection process

Preliminary studies were based on the clinical and electrophysiological evaluation of patients’ tremors to identify the affected muscles (Table 3).

**Table 3:** A review of the approach of the first injection of botulinum toxin in the treatment of Parkinson’s tremor.


AUTHOR, YEAR	INDEX	ECR	ECU	EDC	EPL	EIP	FCR	FCU	FDL	FPL	FDP	FDS	FPB

Jankovic & Schwartz, 1991		✓	✓				✓	✓						

Trosch. R.M, et al., 1994	No.	9	5	2			10	9						

	Dose	11.92 ± 3.84	11.67 ± 3.54	26.67 ± 22.55			18.21 ± 6.68	17.31 ± 6.96						

Pullman, 1996		20.4 ± 14.2	15.8 ± 7.0	16.3 ± 5.5	6.5 ± 5.8	5.8 ± 2.8	25.6 ± 13.2	26.0 ± 16.7	53.3 ± 15.3	19.6 ± 7.2	30.6 ± 8.7	17.6 ± 9.4		

Henderson, 1996		✓	✓	✓	✓	✓	✓	✓	✓	✓	✓			

Rahimi. F. et al., 2013	No.	5	5				4	4		1		1		

	Dose	22 ± 3	22 ± 3				22 ± 3	22 ± 3		10		15		

Rahimi. F. et al., 2015	No.	24	28				24	28						

	Dose	18.5 ± 8.2	16.8 ± 6.7				16.3 ± 7.0	16.8 ± 6.7						

Samotus. O, et al., 2017		✓	✓				✓	✓						

Mittal, S. O., et al., 2017	No.	19	10	18			27	25			7	6	11	

	Dose	5–10	5–10	5–10			10–15	10–20			10	10–20	5–10	

Niemann & Jankovic, 2018							6	6						

Kreiser, 2019														

**AUTHOR, YEAR**	**INDEX**	**PT**	**PQ**	**BICEPS**	**TRICEPS**	**SUP**	**BRACHORADIALIS**	**PECTORALIS MAJOR**	**TERES MAJOR**	**DELTOLID**	**SUPRASPINATUS**	**APB**	**OPPONENS POLLICIS**	**TOTAL**

Jankovic & Schwartz, 1991														1

Trosch. R.M, et al., 1994	No.	1		2	2									12

	Dose	35.00 ± 7.07		63.23 ± 15.28	52.50 ± 23.98									107.50 ± 50.56

Pullman, 1996		22.3 ± 6.2	17.9 ± 4.3	96.7 ± 32.7	58.6 ± 15.7	32.5 ± 14.7	51.4 ± 9.0	67.0 ± 27.3	65.2 ± 18.0	108.1 ± 24.6		3.1 ± 1.3	4.4 ± 1.3	

Henderson, 1996														

Rahimi. F. et al., 2013	No.	4		1	1	4								7

	Dose	21 ± 3		50	50	19 ± 3								97 ± 52

Rahimi. F. et al., 2015	No.	25	25	23	10	22	1	9	6	4	5			28

	Dose	17.4 ± 5.1	16.0 ± 4.9	33.9 ± 10.3	29.5 ± 10.1	17.3 ± 4.5	20.0 ± 0.0	33.3 ± 8.7	25.8 ± 6.7	30.0 ± 9.4	28.0 ± 2.4			174.1 ± 66.8

Samotus. O, et al., 2017		✓	✓	✓	✓	✓	✓	✓	✓	✓	✓			

Mittal, S. O., et al., 2017	No.	25			23	3	5					6	1	

	Dose	10–20			10–15	10	10					5–10	5	

Niemann & Jankovic, 2018														47.9 ± 11.5

Kreiser, 2019														


**Note** All values are presented as mean ± standard deviation. A tick sign indicates that the injection has been given in that muscle. The results are related to the main injection and Booster injection, so in cases where even one person was injected, the indicators could be calculated. **Abbreviations No:** the number of injected people; ECU: extensor carpi ulnaris; ECR: extensor carpi radialis; EDC: extensor digitrorum communis; FCU: flexor carpi ulnaris; FCR: flexor carpi radialis; FDL: flexor digitorum longus; FPL: flexor pollicis longus; EIP: extensor indicisproprius; EPL: extensor pollicis longus; FDP: flexor digitorum profunds; FDS: Flexor digitorum subllmis; FPB: Flexor pollicis brevis; PT: Pronator teres; PQ: Pronator quadratus; SUP: supinator; APB: Abductor pollicis brevis.

In this regard, one study carried out an accurate limb clinical evaluation. The tremor was assessed while overcoming gravity (postural tremor), in purposeful movements (kinematic tremors), and during specific activities such as writing (task-specific tremor). The surface electrode was located on the target limb, and both agonist and antagonist muscles were selected (e.g., extensors and flexors of the wrist). BoNT was injected at four to six different sites anatomically related to the involved muscles in tremor production, comprised of wrist extensors [mainly extensor carpi radialis (ECR) and extensor carpi ulnaris (ECU)] and wrist flexors [mainly flexor carpi radialis (FCR) and flexor carpi ulnaris (FCU)]. BoNT doses for the opposing muscle groups varied depending on the associated dystonia [25].

Another study relied on clinical examination and electromyography (EMG) findings to standardize the selection of muscles and BoNT injections. Investigators evaluated the wrist flexor muscles [such as FCU, FCR, ECR, ECU, pronator teres (PT), supinator, brachioradialis, flexor digitorum superficialis (FDS), extensor digitorum communis (EDC), biceps, and triceps] by surface EMG, and injected the muscles with rhythmic changes. Almost every patient received wrist flexor and extensor injections unless no tremor activity had been detected [26].

In another study, after determining a suitable muscle through clinical judgment and EMG, BoNT was injected only when the least effort with a short increase time (<100 μs) was made to use a few motor units, with no contraction in adjacent muscles. In some cases, especially in the wrist flexors or fingers, BoNT was injected localized, expecting the best functional results. If possible, involuntary muscle activity was distinguished from compensatory activity to prevent wrong injections [21].

Another investigator using clinical judgment performed EMG-guided injections into the extensor muscles [EDC, ECR, ECU, extensor indicis proprius (EIP), and extensor pollicis longus (EPL)] and the flexor muscles [FCR, FCU, and flexor pollicis longus (FPL) and flexor digitorum profundus (FDP)] [27].

The EMG-guided single-stage injection paradigm was used in a pilot study. The most injected muscles included ECU and ECR [22].

Recent research on the use of BoNT formulation has led to the development of a variety of techniques in clinical decision-making and muscle selection for toxin injection. One of the substantial issues is the hand and finger weaknesses after BoNT therapy, which has been significantly overcome following the recently developed methods. Accordingly, a recent paper discussed the Yale method and the whole-arm standardized kinematic tremor assessments as the technology-based injection methods for BoNT-A therapy of essential (ET) and PD tremors [30].

The recently introduced Yale technique advocating extensive EMG screening of forearm muscles before injection (a blinded study) and the TKA technique reported by the Western University (London, Ontario) researchers (several open label, prospective studies) have shown efficacy of BoNT therapy in PD tremor, while causing significantly less weakness (less than 10%) compared to previous studies on tremor. These encouraging results, however, have been challenged recently by the data from two recent retrospective studies.

In the recently introduced Yale technique, the selective injection pattern was used in ET after detecting the tremor-causing muscles in the hand and forearm by extensive EMG screening. Eight often active forearm muscles were screened with comprehensive needle EMG, and only active muscles were injected. The BoNT-A dosage was designated according to muscle size and activity. Tremor response was assessed by Fahn-Tolosa-Marin (FTM) scale, items 20 and 16 of the unified Parkinson’s disease rating scale (UPDRS), and the patient’s global impression of change (PGIC). The improvements in resting tremors were significant at four weeks and eight weeks and in the action/postural tremors at eight 8 weeks after BoNT treatment compared to the placebo. Quality of life improved in most patients, but it did not reach statistical significance. Interestingly, only one participant (less than 10 percent of patients in the treatment group) developed unacceptable wrist extensor weakness, and there were no statistically significant differences regarding hand weakness between the study groups [31]. In another trial at Yale, using the same methodology, moderate weakness of the hand was detected in 13.5 percent of patients [24].

The Kinematic and standardized tremor measures have been explained in recent studies. In this technique, four motion sensors were placed on the whole arm. Participants were requested to perform rest and scripted activity tasks, and motion sensors captured tremor severity in angular root mean square amplitude by electrogoniometer (SG150, Biometrics Ltd) in multiple degrees of freedom in the wrist, elbow, and shoulder joints. Root mean square units are a standard method for tremor intensity analysis, and data of different degrees of freedom from the same joint are combined. Kinematic analysis showed the proportion of the directional involvement and the segmentation of tremors during a particular task. The BoNT-A dosages were determined solely, focusing on each joint separately, using the kinematically measured total tremor amplitude, the injector’s clinical expertise, and therapeutic responses. The participants were followed after three injections, once every 16 weeks, for 48 weeks. A significant decrease in the mean UPDRS-20 was detected at weeks 16 and 32 and in the FTM tremor severity scores at week 6. The bothersome muscle weakness was perceived only in three patients (11 percent) [23].

Another study determined the biomechanical patterns among 52 ET and PD patients undergoing kinematic-guided toxin injection every four months. Motor tracking devices on the forearm, wrist, elbow, and shoulder joints recorded the tremor intensity in the angular root mean square and the degree of freedom. For each participant, the mean tremor amplitude during three experiments per joint was shown as the root mean square degree for each experiment [20].

### Findings on the dose (unit) of local BoNT injection in the target muscles

Summarizing the studies showed that most injections were performed in the ECR, ECU, FCR, FCU, PT, biceps, and triceps. Moreover, the injection dose was usually between 68–100 units of Ona-BoNT/A in each upper limb, distributed between 3 to 6 injection sites. After the initial quantitative tremor analysis, a booster was injected on the same day at 1–6-hour intervals, if needed. The following muscles and doses were routinely injected: FCR 16–25 units; FCU 16–25 units; ECR 10–15 units; and ECU 10–15 units (Table 3). If the muscles were larger (or smaller) than average, or tremors were thought to be driven relatively more (or less) by a particular one, the BoNT dosages would be higher or lower.

### Results of outcomes and criteria evaluated in the studies

The first outcome group included the non-kinematic tremor intensity assessments, the FTM, the National Institute of Health Collaborative Genetic Criteria (NIHCGC) tremor score, and UPDRS. The UPDRS-20 and UPDRS_21 are used to evaluate the level and quality of PD tremors [32].

Analysis showed a decrease in UPDRS_20 and UPDRS_21 indices after BoNT injection in the hands by 1.8 ± 2.5 and 1.2 ± 3.8 units, respectively. For example, tremor intensity decreased from 1.6 ± 0.9 to 0.9 ± 1.0 in one study [23] and from 2.47 ± 1.31 to 1.27 ± 0.97 in another study [24], based on UPDRS_21 (Table 4). The findings did not reach the meta-analysis stage, and their significance was not determined. However, there was a mean reduction in tremor intensity (0.7–1.3 points) within a 1–4-point scoring range.

**Table 4:** The reported indicators related to the outcomes in the articles included in the review.


AUTHOR, YEAR	PRIMARY OUTCOME: CLINICAL SEVERITY OF HAND TREMOR																	

	(CLINICAL SCALES/NON KINEMATIC MEASUREMENT METHOD)																	

	FTM				SPINAL DRAWING		LINE DRAWING		NIHCGC		UPDRS QUESTIONNAIRE HAND ONLY (SCORE 0–4)							

											ITEM 16		ITEM 20			ITEM 21		
						
	PRE. INJ.	POST. INJ (4–6W)	POST. INJ (16 W)	POST. INJ (96 W)	PRE. INJ.	POST. INJ (4–6W)	PRE. INJ.	POST. INJ (16W)	PRE. INJ.	POST. INJ	PRE. INJ.	POST. INJ	PRE. INJ.	POST. INJ (16 W)	POST. INJ (96 W)	PRE. INJ.	POST. INJ (16 W)	POST. INJ (96 W)

Trosch. R. M., et al., 1994																		

Rahimi. F. et al., 2013					1.4 ± 1.5	0.7 ± 1.5	1.0 ± 1.5	0.7 ± 1.5					(20 & 21 item from 8): baseline: 4.8 ± 1.5; post score: 2.1 ± 1.4					

Rahimi. F. et al., 2015	9.6 ± 5.9				1.5 ± 1.5		1.2 ± 1.5						2.7 ± 0.6	2.0 ± 0.8		1.6 ± 0.9	0.9 ± 1.0	

Samotus. O, et al., 2017	9.6 ± 5.9	9 ± 5.7	9.1 ± 4.8	8.6 ± 3.3									2.7 ± 0.6	2.0 ± 0.8	1.3 ± 0.9	1.6 ± 0.9	0.9 ± 1.0	0.8 ± 0.7

Mittal, S. O., et al. (Placebo/IncoA), 2017									3 (2–4)	0 (–2 to 2)	2 (1–4)	0 (–3 to 1)	3 ± 0.64	0 ± 0.63*		1.73 ± 0.98	0 ± 1.93*	

Mittal, S. O., et al. (IncoA/Placebo), 2017									3 (2–4)	1 (–1 to 2)	3 (1–4)	0 (0 to 2)	3.27 ± 0.36	1.53 ± 1.31*		2.47 ± 1.31	1.27 ± 0.97*	

**AUTHOR, YEAR**	**SECONDARY OUTCOME: KINEMATIC EVALUATION OF HAND TREMOR**																	

	**KINEMATIC EVALUATION**																	

	**TREMOR COMPOSITION (TORSIOMETER)/INCLINOMETER (DEGREE)**				**ACCELEROMETER (M/S^2^)**													

	**REST**		**POSTURE**		**REST**		**POSTURE**											
			
	**PRE. INJ.**	**POST. INJ**	**PRE. INJ.**	**POST. INJ**	**PRE. INJ.**	**POST. INJ**	**PRE. INJ.**	**POST. INJ**										

Trosch.R.M., et al., 1994					30.3 ± 15.7	23.1 ± 13.6	20.9 ± 14.7	16.5 ± 11.2										

Rahimi. F. et al., 2013	0.9 ± 1.2		1.2 ± 1.8		1.5 ± 2.1		2.3 ± 2.7											

Rahimi. F. et al., 2015																		

Samotus. O, et al, 2017																		

Mittal, S. O., et al. (Placebo/IncoA), 2017																		

Mittal, S. O., et al. (IncoA/Placebo), 2017																		


**Abbreviations** FTM: Fahan-Tolosa-Martin tremor scale; NIHCGC tremor severity: National Institutes of Health Collaborative Genetic Criteria; UPDRS: Unified Parkinson’s disease rating scale; Inj.: injection.

The second outcome group, the kinematic evaluation of tremor severity, was measured by installing a sensor device on the affected hand and performing evaluations with a torsiometer, inclinometer, accelerometer, and electro-goniometer.

The third outcome group focused on patients’ pain intensity, daily functioning, and quality of life. Pain intensity was evaluated using a visual analog scale (VAS), the ability to perform activities of daily living was measured using daily life activities questionnaire, muscle strength was measured using a handheld dynamometer or ergometer, perceived weakness using a Likert scale, and quality of life was measured using Parkinson’s Disease Questionnaire-39 (PDQ-39), the Quality of Life in Essential Tremor Questionnaire (QUEST), or PGIC scales.

### Results of therapeutic efficacy of local injection of BoNT on the outcomes of the first group

**Figure 2:**
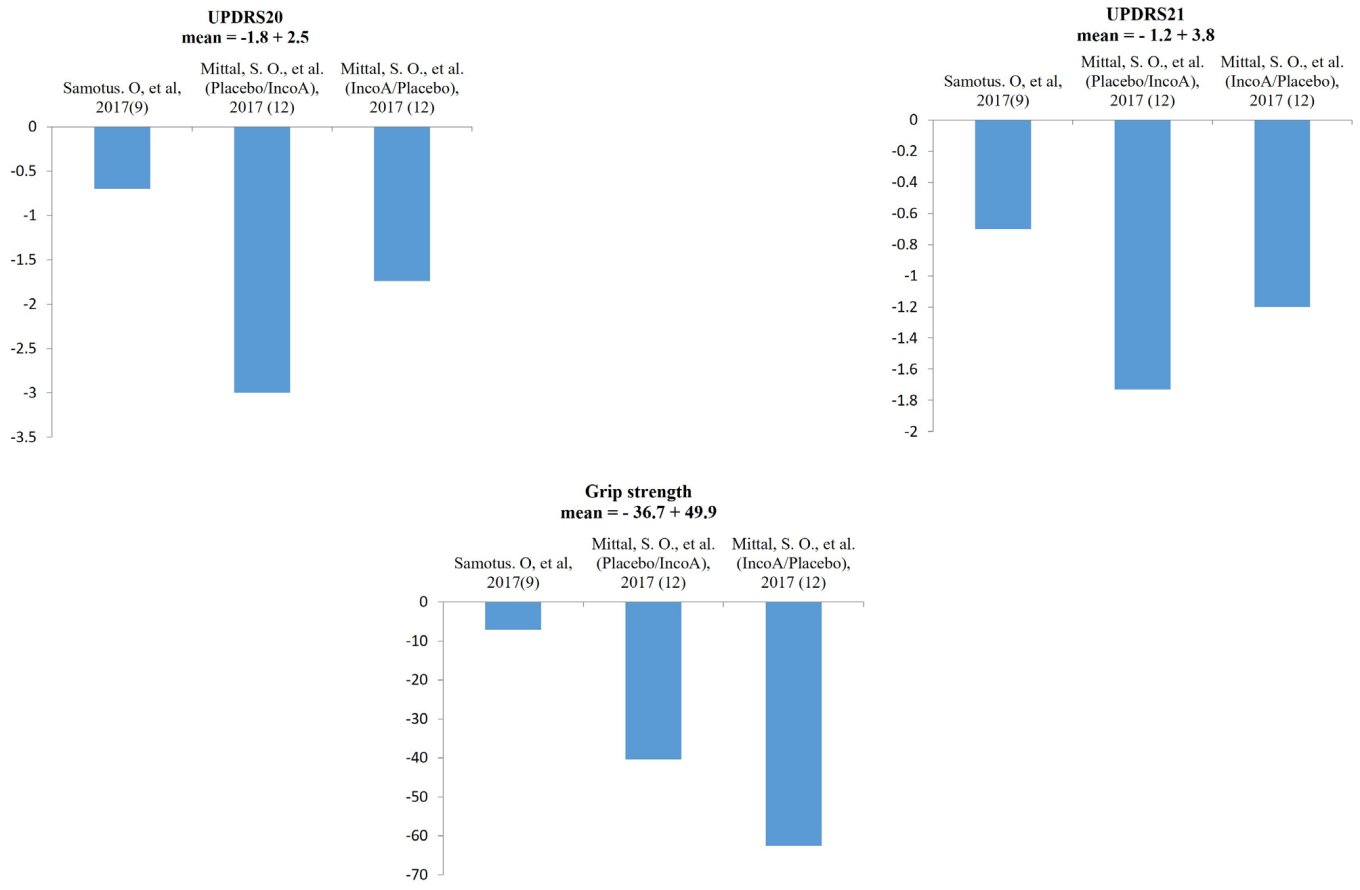
The therapeutic efficacy of local injection of botulinum toxin. The columns represent the changes on Unified Parkinson’s Disease rating scale-20, Unified Parkinson’s Disease rating scale-21, and grip strength before and after the injection.

After the BoNT injection, the decrease of the mean UPDRS_20 score differed from 0.7 ± 0.72 units to a maximum of 3.0 ± 0.64 units among the studies. The mean UPDRS_20 score improvement was 1.8 ± 2.5 units (Figure 2, Part 1). The decrease of the mean UPDRS_21 score differed from 0.7 ± 0.95 units to a maximum of 1.73 ± 1.67 units among the studies. The mean UPDRS_21 score improvement was 1.2 ± 3.8 units (Figure 2, Part 2). The mean grip strength score changes differed from 7.1 ± 8.6 units to a maximum of 62.2 ± 22.3 units among the studies. However, an increase of 10.35 ± 0.54 units in grip strength was seen in one study after the BoNT injection. The mean change in the patient’s grip strength due to the BoNT injection was 36.7 ± 49.9 units (Figure 2, Part 3).

### Side effects and contraindications

The BoNT side effects are partly due to the unintended release of the toxin to adjacent muscles or structures that may cause muscle weakness [33]. Contraindications for BoNT treatment include pregnancy, a known neuromuscular transmission disorder and myopathy, and a history of muscle weakness. The most common side effects are dose-dependent and occur at the injection site. Systemic adverse events are rare and usually occur after a BoNT injection of a ten times higher dosage than usual [34].

In many of the discussed studies, there was a high prevalence of dose-dependent side effects, including finger weakness and pain at the injection site. In many older studies, patients had self-reported excessive weakness. This was improved in recent studies by focusing on the target muscles.

## Discussion

Considering the limited effectiveness of oral pharmacotherapy in many tremor disorder managements and the ineligibility of many PD patients for surgical interventions, there is an urgent need for alternative treatment options. However, there are only a few recommendations for using a specific dose and method of BoNT-A injection to treat PD patients. This study provides evidence of significant improvement in PD tremor in short- and long-term follow-up assessments after BoNT-A injections. BoNT injection administered in a personalized approach based on distribution, amplitude, and other features of the tremor would be a good option in PD patients with troublesome tremors.

The results showed an improvement in tremor intensity according to the UPDRS_20 and UPDRS_21 indices by 1.8 ± 2.5 and 1.2 ± 3.8 units after customized BoNT injection, respectively, with a mean reduction of 0.7 to1.3 points within a 1–4-point scoring range. A high prevalence of dose-dependent side effects, such as finger weakness, is seen in older studies which have been significantly overcome by methods such as Yale and technology-based BoNT-A injection methods introduced in recent years. Although a standard protocol is easy to repeat, the fixed injection method is unlikely to benefit all patients because of the different upper limb tremor amplitude and levels from person to person. There is growing evidence for a customized approach, and the various studies mentioned earlier have many similarities regarding muscle selection and dose approach. However, there is no guideline to instruct other physicians who may wish to start treating their patients.

### Initial evaluation

Most upper limb tremors are of the following types: forearm pronation/supination (P/S) tremor, wrist flexion-extension (F/E) tremor, shoulder internal-external rotation (I/E) tremor, and elbow F/E tremor.

The patient’s body structure, muscle size, and tremor force provide evidence to help adjust the dose. The initial dosage can vary considerably but typically ranges between 30–100 units of Ona-BoNT/A and 100–300 units of Abo-BoNT/A distributed to each upper limb at 3 to 6 injection sites [20,35]. The Ona-BoNT/A usual dosage is 25 units for FCR and FCU muscles and 15 units for ECR and ECU [10,21].

In the muscle selection process for BoNT injection in any tremor type, the following items should be taken into account: [35]

1. Forearm P/S tremor: In this case, PT, and supinator quadratus (SQ), and pronator quadratus (PQ) muscles are always treated. Caution should be exercised when injecting into the SQ because it requires passage through the EDC. Before the injection, the patient is asked to extend the middle finger to ensure no needle in the muscle to prevent finger drop. The PQ is accessible through the dorsal surface of the distal forearm muscle through the interstitial membrane.
2. Wrist F/E tremor: generally, two flexors (FCR and FCU) and two extensors (ECR and ECU) are treated. We inject into the most superficial part of forearm flexors, especially the FCR, to prevent the FDS muscle from affecting and subsequent grip power weakening.
3. Shoulder I/E tremor: Higher doses can be injected to treat these muscles. In obese patients, accessing the teres major and teres minor muscles may be difficult. Further, caution must be taken to avoid perforation in the intercostal space and potentially pneumothorax, especially in lean patients. Usually, pectoralis major and deltoid muscles are not injected due to shoulder weakness probabilities.
4. Elbow F/E tremor: Substantially lower doses are injected into these muscles (especially the biceps) due to the probabilities of elbow flexion weaknesses and dysfunction. In the cases of no prominent tremor, injection of the biceps is often delayed.

Ideally, the patients should be followed up for 4 to 6 weeks to determine the maximum effect of the injection and the need for further sessions. The patients are asked about focal weakness or checked with a hand dynamometer.

The most common BoNT injection side effects are related to the injection site, and systemic effects are rare [36]. In many of the discussed studies, there was a high prevalence of dose-dependent side effects, such as finger weakness and pain at the injection site. Because BoNT works directly at the neuromuscular junction, the exact localization of the motor endplate increases the injection clinical response by about 50 percent. However, visually determining the target muscle and precise location remains challenging. Hence individualization of BoNT-A therapy using a needle EMG and some kinematic approach could provide either experienced or novice injectors with standardized and user-friendly tools [37]. These approaches provide more accurate BoNT injection and minimize the possible dissemination of toxins and neutralization by antibody formation. Moreover, focusing the toxin injection on the flexor muscles and avoiding extensor muscles allows the patients to maintain finger grip strength, further improves the patient’s life quality, and the return of arm function in daily living activities such as writing and eating. The limitation of the Yale technique is the pain and discomfort related to needle insertion into the muscle. In addition, multiple needles cannot be inserted into all the muscles simultaneously for matched recording. However, in experienced hands, the screening and injections can be done in less than one hour using a hand-held EMG. The pain and discomfort are overcome in the Kinetic/sensor-based method. Although the kinematic technique can record and analyze the tremor concurrently from the whole arm, it cannot give the same reliability of information on individual muscle activities as the Yale method. The kinematic instrument is expensive (the current unit costs about $10,000) and is not yet on the market. A future comparator study can determine which method is better regarding efficacy, cost of the instruments, analysis fee, cost-benefit, and the time needed for each to provide acceptable clinical consequences.

Furthermore, the injection into any muscle that contracts involuntarily in a part of the tremor syndrome is impossible due to the required total BoNT dosage and the undesirable weakness. The fact that even limited injection into only two forearm flexor muscles (FCU and FCR) has had favorable results in improving the tremor indicates that the mechanism of action of the toxin is probably more than that of local muscle weakness solely and the central effects may be involved in this process through the modulation of intracortical excitability and widespread inhibition of shivering muscle activity.

## Study Limitations

Considering the lack of sufficient and homogenous data, the reported values of the effectiveness of BoNT injection were presented descriptively based on quantitative indicators. Additionally, a review study depends on the findings of existing published studies, so any limitations of the included studies are inevitable limitations of this study. For example, the tremor etiology was unclear in most studies. The co-existence of essential or dystonic tremors with PD should be distinguished when treating hand tremors.

Another limitation is the number of PD patients with postural, action, or resting tremors was not specified separately among primary studies. However, in one study, tremors were evaluated in static tasks, rest, postural and functional tasks, kinetic, and weight-holding tremors [31]. In two studies, the trials were performed in two rest (forearm supported in lap and forearm supported in a board) and two postural (arms pronated with palms down in one study and arms semi-supinated with palms facing each other in the other one) positions [16,33]. Two studies showed a significant reduction in rest and action tremor based on UPDRS_20 and UPDRS_21 and the FTM rating scale at 4 and 8 weeks after toxin injection [24,28]. Nonetheless, considering the limited studies in assessing the efficacy of BoNT injection in PD patients, this study could not conclude tremor-specific results. Hence, further clinical trials are suggested based on tremor types, such as rest, postural, and kinematic, to identify the best responders for the implemented treatments.

## Suggestions for future studies

This systematic study aimed to present an evidence-based approach for BoNT injection in large-scale clinical trials that are critically needed in this area. Overall, due to the heterogeneity of studies in terms of injection and evaluation methods, it is recommended that a randomized clinical trial with a larger sample size be conducted to enrich the articles and reduce errors and bias.

Considering that local BoNT injection has a cumulative effect because of the toxin nature and considering one of the previous studies [20], which included six injections every 16 weeks, the multiple BoNT injection effects should be compared with that of the single injections in future studies.

The medical literature is scarce regarding the effectiveness of simultaneous treatment of PD tremors and occupational therapy with local BoNT injection and should be considered in future study designs. It seems that it is better to apply occupational therapy and local BoNT injection in the case group to determine whether it improved the effectiveness of local BoNT injection [6]. Some studies have been published on the efficacy of concomitant occupational therapy and local BoNT injection in other conditions. Recently a systematic review has measured the effectiveness of BoNT treatment for upper limb spasticity after stroke [38].

Inco-BoNT/A was injected into the forearm muscles and lumbricals meanwhile in one of the discussed studies [24]. Upper limb tremor in PD patients is mainly in the hand and distal forearm area, and the most frequent tremor, is a rest tremor around the metacarpophalangeal joints of the hand. Most of the movement in the metacarpophalangeal joints is done by the lumbricals and interossei. So, future studies should focus on local BoNT injection in these muscles and investigate its positive effects versus adverse effects.

As a last suggestion, the efficacy of other powerful techniques, such as sonography before injection to describe forearm muscles participating in tremors, is needed to be examined by future controlled studies.

In conclusion, a comprehensive review of studies showed that local BoNT injection could be considered an adjunctive therapy without serious side effects for PD patients who have not improved with medication or whose drug side effects are difficult to tolerate. Muscle kinematic and EMG-guided evaluations and confirmation with electrical stimulation allow for more accurate localization and minimize the possibility of toxin diffusion and antibody formation. Overall, due to the heterogeneity of studies in terms of injection dosages and sites, and evaluation methods, randomized controlled clinical trials with larger sample sizes and higher follow-up periods are suggested to allow more accurate analyses and conclusions.
